# Nontuberculous mycobacterial infection in a clinical presentation of Fitz-Hugh-Curtis syndrome: a case report with multigene diagnostic approach

**DOI:** 10.1186/1472-6874-14-95

**Published:** 2014-08-12

**Authors:** Hang-Yong Jang, Peter D Burbelo, Yang-Seok Chae, Tak Kim, Yunjung Cho, Hyun-Tae Park

**Affiliations:** 1 Department of Obstetrics and Gynecology, Korea University College of Medicine, 126-1, 5-ga Anam-dong, Seongbuk-gu, Seoul 136-705, South Korea; 2 Dental Clinical Research Core, National Institute of Dental and Craniofacial Research, National Institutes of Health, Bethesda, Maryland, USA; 3 Department of Pathology, Korea University College of Medicine, Seoul, South Korea; 4 Department of Laboratory Medicine, Korea University College of Medicine, Seoul, South Korea

**Keywords:** Nontuberculous mycobacteria, Fitz-Hugh-Curtis syndrome, Laparoscopy

## Abstract

**Background:**

Fitz-Hugh-Curtis syndrome (FHCS) is caused by inflammation of perihepatic capsules associated with pelvic inflammatory disease. In recent years, infections with nontuberculous mycobacteria (NTM) have been increasingly occurring in immunocompromised and immunocompetent patients. However, NTM has never been reported in patients with FHCS. We present the first case of a patient with extrapulmonary NTM infection in a clinical presentation of FHCS.

**Case presentation:**

A 26-year-old Korean woman presented with right upper quadrant and suprapubic pain. She was initially suspected to have FHCS. However, she was refractory to conventional antibiotic therapy. Laparoscopy revealed multiple violin-string adhesions of the parietal peritoneum to the liver and miliary-like nodules on the peritoneal surfaces. Diagnosis of NTM was confirmed by the polymerase chain reaction analysis results of biopsy specimens that showed caseating granulomas with positive acid-fast bacilli. Treatment with anti-NTM medications was initiated, and the patient’s symptoms were considerably ameliorated.

**Conclusions:**

An awareness of NTM as potential pathogens, even in previously healthy adults, and efforts to exclude other confounding diseases are important to establish the diagnosis of NTM disease. NTM infection can cause various clinical manifestations, which in the present case, overlapped with the symptoms of perihepatic inflammation seen in FHCS.

## Background

FHCS is characterized by inflammation of perihepatic capsules and adhesions between the liver capsule and anterior abdominal wall with concomitant pelvic inflammatory disease (PID). The diagnosis of FHCS can be difficult because its symptoms and physical findings can mimic those of many other diseases. FHCS is considered in women with right upper quadrant (RUQ) pain related to hepatobiliary diseases such as cholecystitis, cholelithiasis, and hepatitis and in women with pulmonary-related diseases such as pleurisy, pulmonary embolism, and pneumonia. FHCS may also be present in patients with perforated ulcer, subphrenic abscess, pancreatitis, appendicitis, or herpes zoster [1]. It is most frequently caused by the sexually transmitted pathogens of *Chlamydia trachomatis* and *Neisseria gonorrhoeae*. However, genital or extrapulmonary mycobacterial infection is often neglected during evaluation for suspected FHCS [2]. Only a few studies have reported (Mycobacterium tuberculosis) MTB-related FHCS, and no reports of NTM in patients with FHCS have been published. Herein, we present the first reported case of a patient with extrapulmonary NTM infection in a clinical presentation of FHCS.

## Case presentation

A 26-year-old Korean woman presented with RUQ pain for 2 weeks, and more recently, intermittent suprapubic pain. She had a history of emergency appendectomy a year ago at a local clinic. Otherwise, she had no chronic medical illness or prolonged use of medicine. On physical examination in the emergency department, the abdominal wall was soft but produced RUQ tenderness to pressure over the liver. A pelvic examination revealed cervical motion tenderness without adnexal tenderness. A transvaginal sonogram revealed a small fluid collection in the pouch of Douglas but an otherwise normal appearance. Vaginal and cervical swab cultures were positive for *Escherichia coli*. Her laboratory values were all within the reference ranges, except for an elevated C-reactive protein (CRP) level (6.1 mg/dL). Serological screening showed negative results for the surface antigen of the hepatitis B virus (HBsAg), anti-hepatitis C virus, anti-human immunodeficiency virus (HIV), and venereal disease research laboratory test. Chest radiographic findings were normal. An abdominopelvic contrast-enhanced computed tomographic (CT) scan showed a small collection of pelvic fluid, diffuse omental-mesenteric infiltration, and hepatic capsular enhancement over the medial segment during the early phase, which non-definitively suggested FHCS. Abdominal ultrasonography showed that the liver, kidneys, spleen, and pancreas were of normal size, without increase in echo patterns; furthermore, the gallbladder was normal, without focal lesions or biliary tree dilatation. After FHCS with PID was diagnosed, treatment with empiric broad-spectrum antibiotics was initiated. At the patient’s scheduled follow-up visit after 2 weeks of antibiotic therapy, the intermittent RUQ pain had persisted but with improvement of symptoms.

**Figure 1:**
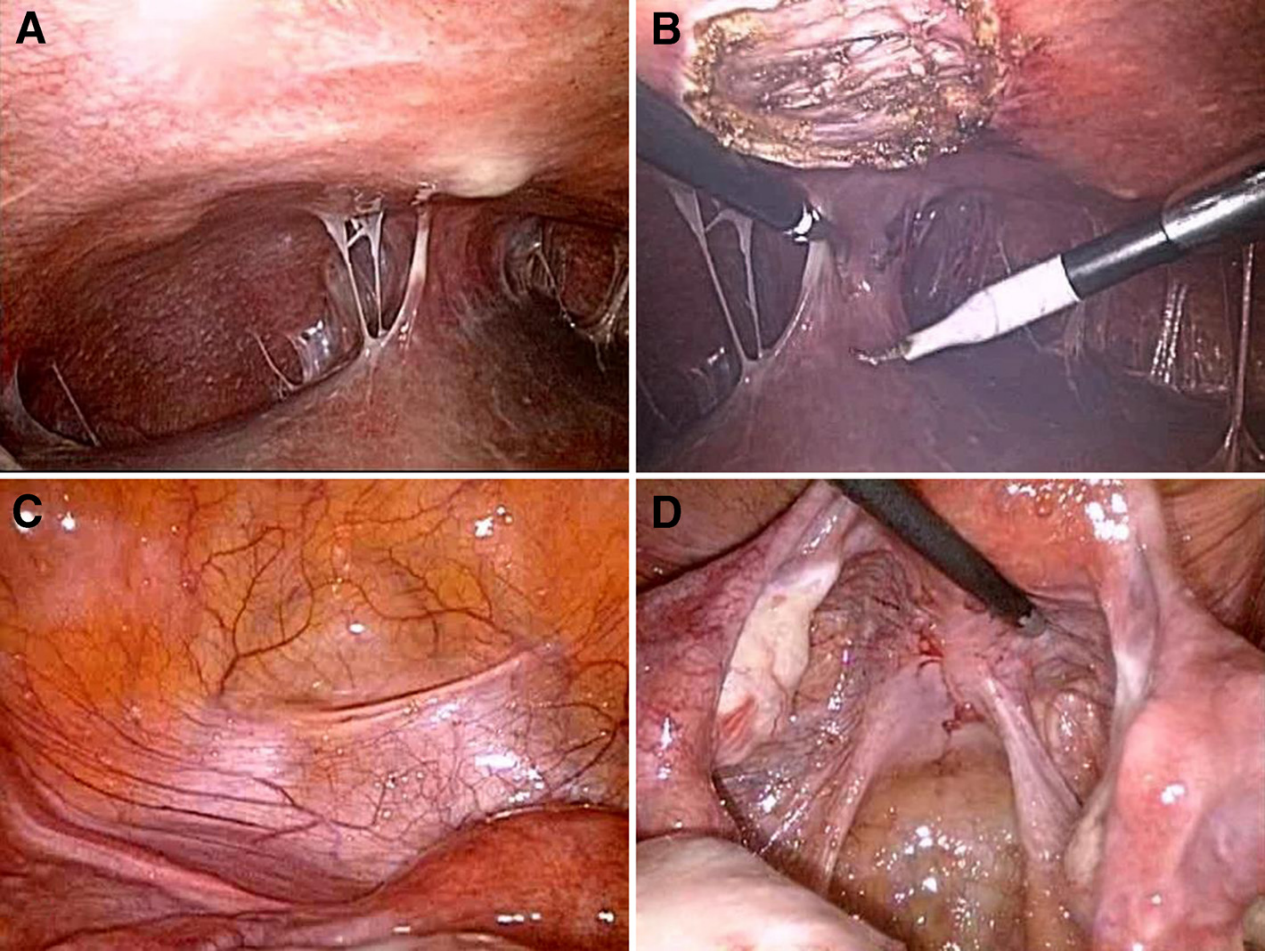
Laparoscopic views of the peritoneal cavity revealed the formation of large nodules on the anterior parietal peritoneum, which were completely excised and showed NTM by PCR analysis, multiple membrane and violin-string adhesions of the parietal peritoneum to the liver (A and B), and military-like nodules in the surfaces of the anterior pelvic peritoneum, uterus, and pouch of Douglas (C and D).

**Figure 2:**
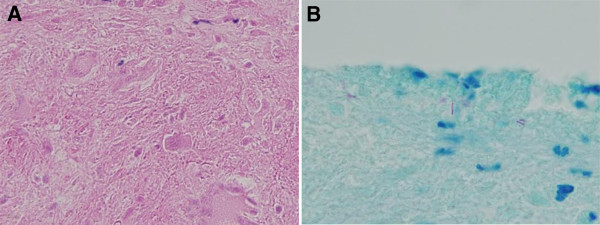
Histopathological examination revealed necrotizing granulomatous inflammation (A) with positive acid-fast bacilli on Ziehl-Neelsen staining (B).

Three months later, she was readmitted with severe RUQ colicky pain with a rapidly progressive onset, which was exacerbated by deep inspiration. Her laboratory values were all within the reference ranges, except for an elevated CRP level (35.9 mg/dL). She underwent a diagnostic laparoscopic procedure, which revealed multiple violin-string adhesions between the liver and anterior abdominal wall and miliary-like nodules on the surfaces of the anterior peritoneum, uterus, and pouch of Douglas (Figure 1). We collected the specimens using laparoscopic equipment subjected to automated sterilization with vaporized hydrogen peroxide (STERRAD 100S Sterilization System, Johnson & Johnson, USA) in a highly sterile operation room with air shower to avoid cross-contamination. Histopathological examination revealed caseating granulomas with positive acid-fast bacilli (AFB) on Ziehl-Neelsen staining, which is a strong evidence for the presence of mycobacteria in the specimens (Figure 2). For the differential diagnosis between MTB and NTM, we used a nested PCR protocol (NTM&MTB PCR kit for paraffin-embedded tissue, BioSewoom Inc., Seoul, Korea), which amplifies a fragment of the MTB-specific IS6110 primer and 16S rRNA region of mycobacteria and has approved by the Korea Food & Drug Administration. The patient was repeatedly confirmed by PCR to have NTM, but MTB was not detected, indicating an extrapulmonary NTM infection. For identification of the causative NTM species, PCR-directed sequencing of the 16S rRNA gene and 16S-23S rRNA internal transcribed spacer (ITS) was performed, followed by a basic local alignment search tool (BLAST) sequence analysis. Four NTM species, *M. indicus pranii, M. intracellulare*, *M. chimaera*, and *M. avium,* showed 95% nucleotide identity in the current BLAST database.

Considering that NTM infection is rarely reported in immunocompetent patients, we further extended our study to investigate the patient’s genetic susceptibility to mycobacterial infection and immunological status. To evaluate the genetic defects that predispose susceptibility to mycobacteria, interferon-ɣ receptor 1 (*IFNGR1*), interferon-ɣ receptor 2 (*IFNGR2*), and signal transducer and activator of transcription 1 (*STAT1*) genes were analyzed. PCR was performed with ABI 2720 using primers designed to exons and exon-intron boundaries (Table 1). Direct sequencing of amplified products was performed using ABI 3130 (Applied Biosystems, Foster City, CA). Sequences were analyzed using the Sequencher version 4.10 software (Gene Codes Corporation, Ann Arbor, MI). Four different single-nucleotide polymorphisms (SNPs) were found in the *IFNGR1*, *IFNGR2*, and *STAT1* genes (Table 2). A highly quantitative antibody profiling technology, luciferase immunoprecipitation systems (LIPS), was used to investigate the possibility of whether anti-cytokine autoantibodies, including IFN-α, IFN-γ, granulocyte-macrophage colony-stimulating factor (GM-CSF), interleukin (IL)-12 p35, IL-6, and tumor necrosis factor α, were involved in the opportunistic infection in the patient. However, no detectable anti-cytokine autoantibodies were found in the patient. We also investigated the association between HLA class II genes and the susceptibility to NTM infection. The patient’s HLA-DR/DQ types determined by PCR sequence-based typing were DRB1*0201, DRB1*0302, DQB1*0201, and DQB1*0302. The peripheral blood lymphocyte subsets were within the normal limits, and the fluorescent antinuclear antibody (FANA) test result was negative.

**Table 1:** Primer information

**Gene**	**Exon**	**Forward**	**Reverse**	**Amplicon size (bp)**
IFNGR1	1	CGGTGACGGAAGTGACGTA	AACCGACGAGTTCAAACCAC	372
	2	GGCAATGTGGCATCTTACAA	TGGGAAGGCTGATGAAAGAA	292
	3	CTTTCTCTGGCGTCTCCATC	TTTGGAGCATTCTGGGTTTC	495
	4	TGGTTTAAATGTGGTCCTGCT	TGATCTGTGAGTCTTGCTTGAA	427
	5	TGCATAGTATCGTGCTGTGTTG	TGCAAATGAGTTTGCTTTCC	453
	6	CCTAACTTCTGCTTCCTTCAGTG	GGCAGGTGACATCTTGTTGA	389
	7-1	GCCATTTGGTGGTCCATTAC	GGAGGTGGGGGCTTTTATTA	599
	7-2	GCAGCCATCTGACTCCAATAG	TCACTCCATTTGGTTGATACC	489
IFNGR2	1	GAGCCGAATCCCCTCCAC	TGATCTGAGCACTCCGCATA	401
	2	TGCCATTTCTGCACTTTGAC	AACCCGTGTGGTTAGACAGC	496
	3	GGGTGACAGAGCAAAACTCC	TTTAGGGTGACAGGCAGGAC	482
	4	TCGCACAACTGCACTCTAGC	CTCAGCCTCTATGGGGACAC	489
	5	TCTGAGACCTCACAGCCAGA	CTCATAAGCATTCCCCGATT	473
	6	TAAGCAGCATGGATGGAACA	AAAATTAGCAGGCCATGGTG	429
	7	CCAGCCTAGGCAAGAGTAAGA	CAGGGACAGGCCTTTTAGAG	398
STAT1	3	TGTGTTTCGTGGAGGAGAAA	GTAAACATGGCCCCAAGTCA	422
	4	CTGCTGCTAAAAGCAATGTCA	TGTGCTCAATTGTATTTGCTGA	392
	5	CCCGGAGAATATAAACAAATTCA	CTCCCACTTCTTGGGGCTAT	327
	6	ACAGTTTGCCAGCATTTTCC	GAGGTTTACACCCCAAGCAA	351
	7	CGTGTTTCTCTGGGTTCCAT	TAAAAGTCGGGGATGCTGTT	405
	8	GACTACAGGCGTCTGCCACT	TCATTCTCAACTGGGGCTCT	448
	9	GAGCCCCAGTTGAGAATGAA	TTCCAAGGGAGTGTTTCCAG	420
	10	CCATGATTCCCTTTAATCCAG	TTAAACCCTTGTAAATCATCTGAA	323
	11	GATCGTACTCTCTAACCCCCTTT	GCATTACATTTATGATTGGAAAAA	439
	12	CCTAAAACTGGAGGGGGAGT	AAATCTGCTGGGGTCATTTG	410
	13	GGCTGCAAATCCATAGGTGT	GCCCAAAATGTTGAACTTCC	285
	14	TGCAGAGATGTGAATGAGAGAAA	CAGTCGTGGGATAAGGAGGA	407
	15	GCCAATTTGTCCCATGTTCT	CAAATACCCAGCTCCTTTGC	324
	16	TGTGGCCTGTTGCAATGTTA	CATCAGCGATCTCAACTGGA	400
	17	TCCCCTGCTTTTAGTTGAGG	ACTGCCCTAGCTTACCCACA	410
	18	CCCCTACTGTGAAAGCACCT	TAGCAGAGGGGAAAAGAGCA	348
	19	CCATTTAAATTCCGCCCTCT	CCTTGGCCAGAGAAGAACAC	382
	20	TGTGCTGAATGGGACAGTTC	CTGAAGGGGCAGCCTATAAA	357
	21	CATTCCAGCCATTTTCTTGG	TTCATGTCCCAAACGCACTA	340
	22	CACTCAAATCCATCAACTCTGC	TCGAAAGCAAAACACTGCAT	377
	23	TGACTCAGGCAGGGAGGTTA	TTCAGCTGTGATGGCGATAG	351
	24	CACATCCCTGGAGTGTGAAA	CTCCAACTTGTCATGGCTCA	481
	25	CCAGTGGGCAGCAGAGTAAT	TGCTGGCCTTTCTTTCATTT	362

**Table 2:** Analysis results of genes and single nucleotide polymorphisms (SNPs)

**Gene**	**Name**	**Chromosome Location**	**SNP database ID**	**Amino acid change**
IFNGR1	Interferon, gamma, receptor 1	6q23	rs11575931	-
IFNGR2	Interferon, gamma, receptor 2	21q22	rs2070385	p.Gln64Arg
IFNGR2	Interferon, gamma, receptor 2	21q22	rs11910627	-
STAT1	Signal transducer and activator of transcription 1	2q32	rs2066804	-

For the NTM infection in the patient, the first-line *Mycobacterium avium* complex (MAC) drugs, including clarithromycin, ethambutol, and rifampicin, were initiated. After 6 weeks of taking the medications, even though the CRP level decreased to 1.08 mg/dL, the patient still complained of RUQ pain, which was aggravated by deep inspiration. After 12 weeks of taking the medications, the patient showed a significant improvement in symptoms and resumed normal daily activities. An abdominopelvic enhanced CT scan revealed that ascites had vastly decreased, with improved diffuse omental-mesenteric infiltration and a decrease in the size of the perihepatic space tissue lesion.

## Conclusions

The present patient was an immunocompetent, previously healthy woman without known parental consanguinity or genetic disorders. NTM does not commonly cause disease, but under certain circumstances, these mycobacteria can cause serious morbidity and mortality. Disseminated and extrapulmonary NTM infections are typically associated in several clinical manifestations, including patients with HIV infection who have CD4 lymphocyte counts <100/μL, patients with immunocompromised conditions such as metastatic cancer, patients undergoing organ transplantation, and patients receiving immunosuppressive agents [3]. However, cases of non-HIV infection with disseminated NTM infection have been reported with increasing frequency over the last 10 years, particularly in Asian countries [4,5].

In the present patient, the source of extrapulmonary NTM infection and route of transmission in the abdominal cavity was suspected to be an iatrogenic cause from the prior surgery with host immunological or genetic predisposition with susceptibility of mycobacterial infection. But, no significant autoantibodies were detected against six different cytokines, including two targets, IFN-ɣ and GM-CSF, as previously detected in patients with NTM infection in Taiwan and Thailand [6]. Genetic defects that predispose patients to susceptibility to mycobacteria, known as Mendelian susceptibility to mycobacterial disease, occur in the genes associated with the mononuclear phagocyte and T-helper cell type 1 pathway, including *IFNGR1*, *IFNGR2*, *STAT1*, *IL-12 p40*, and IL-12 receptor β1 chain [7]. We investigated the *IFNGR1*, *IFNGR2*, *STAT1,* and HLA class II genes, and found four different SNPs and HLA-DR/DQ types. A few studies showed positive associations of NTM infection with SNPs and specific allele types of HLA [8,9]. However, these associations are not conclusive owing to the insufficient sample size of the studies and genetic diversity between ethnicities. Therefore, our results require further study for proper elucidation.

For identifying causative NTM species in the patient, PCR-directed sequencing of the 16S RNA gene and 16S-23S rRNA ITS was performed. However, it had limitations due to formaldehyde-induced DNA strand breaks on formalin-fixed specimens and insufficient nucleotide differences between closely related genes in *Mycobacterium* species [10]. Because of degradation, amplification of microbial DNA from formalin-fixed paraffin-embedded tissue is challenging, and no single target gene, such as 16S rRNA, hsp65, rpoB, and sod, can accurately distinguish all *Mycobacterium* species [10-12]. The comparative analysis of 16S rRNA sequence of *M. indicus* pranii (MIP) by using the BLAST sequence analysis at NCBI showed a similarity of more than 99% with other closely related mycobacteria: *M. intracellulare*, *M. arosiense*, *M. chimaera*, *M. seoulense*, *M. avium* subspecies hominissuis, *M. avium* subspecies paratuberculosis, and *M. bohemicum*. Further analysis with rpoB and hsp65 gene sequences resolved the phylogenetic relationship between MIP and other closely related mycobacteria [13]. Multigene sequence-based typing using several gene fragments, including the 16S rRNA, hsp65, rpoB, sod, and/or 16S-23S rRNA ITS sequences, showed significant increases in the power of discrimination and accurate species identification in the genus *Mycobacterium*[10,14].

In this case, four NTM species, *M. indicus pranii*, *M. intracellulare*, *M. chimaera*, and *M. avium*, were matched by 95% in the current BLAST database. Among them, *M. avium* and *M. intracellulare* are the pathogenic mycobacterial species that are the most common NTM species that cause diseases in humans worldwide. In the epidemiological studies of NTM in Korea, MAC followed by *M. abscessus* complex made up most of the NTM species (88%) isolated from patients who tested positive for NTM; this finding suggests that MAC was the causative NTM species in the patient [14]. On the other hand, *M. indicus pranii* and *M. chimaera* are generally considered as non-pathogenic mycobacterial species and have never been reported in Korea. NTM species that are typically not pathogenic and are usually isolated because of contamination include *M. mucogenicum, M. gordonae, M. terrae* complex, *M. scrofulaceum, M. simiae*, and *M. lentiflavum*; these were not found in our specimens [15].

Although it is difficult to differentiate between FHCS and *Mycobacterium*-related peritonitis on laparoscopic examination, the finding of “violin string-like adhesions” is considered a characteristic of FHCS; however, “membrane-like adhesions with multiple small and white nodules” is considered a characteristic of tuberculous peritonitis [16]. In our case, characteristics of hepatic capsular adhesions were found in combination with membrane-like and violin string-like adhesions with multiple large and small white nodules in the liver and abdominopelvic peritoneal surfaces; this finding may help differentiate *Mycobacterium*-related peritonitis from FHCS under laparoscopic observation.

In the present patient, NTM diagnosis was delayed mainly because of the lack of awareness of the clinical manifestations of the disease, but nonspecific clinical presentations of NTM infection and similarities in the clinical characteristics related to FHCS associated with PID also contributed the late diagnosis. The minimum criteria for the diagnosis of PID, as outlined by the Centers for Disease Control and Prevention include lower abdominal, adnexal, and cervical motion tenderness. Empirical treatment is recommended for sexually active women, unless another cause for the clinical signs can be identified despite relatively high rates of false-positive and false-negative PID diagnosis [17]. In the present case, the treatment of extrapulmonary NTM infection was delayed until a biopsy-proven diagnosis of NTM infection by PCR analysis was made after diagnostic laparoscopy. Early diagnosis and treatment, especially of severe NTM infection, are crucial for improving prognosis and reducing morbidity and mortality. Therefore, patients refractory to empiric antibiotic therapy without a progressive decrease in levels of inflammatory markers and who have negative cervical or vaginal culture results need to be promptly evaluated further to determine the cause of pain. Diagnostic laparoscopy can be useful for diagnosis of intra-abdominal lesions and adhesions. Localized lesions and adhesions can also be treated using endoscopic resection with antibiotic adjuvants for symptom control and treatment.

In conclusion, establishing the diagnosis of NTM infection requires the awareness of NTM as potential pathogens, even in previously healthy adults, and efforts to exclude other confounding diseases. NTM infection can cause a wide range of clinical manifestations, including, as observed in the present case, severe adhesions and perihepatic inflammation that overlapped the symptoms of FHCS.

### Consent

Written informed consent for publication of this case and any accompanying image was obtained from the patient. A copy of the written consent is available for review by the Series Editor of this journal.

## Competing interests

The authors declare that they have no competing interests.

## Authors’ contributions

HTP and TK were involved in the direct clinical care (diagnosis, decision making, and treatment) of the patient in the reported case. YSC was involved in the histological and microbiological investigations. PDB was involved in the molecular investigation of the anti-cytokine autoantibodies. YC was involved in the genetic and immunologic investigations of Mendelian susceptibility to mycobacterial disease. HYJ, PDB, YC, and HTP conducted the literature search and drafted the manuscript. All the authors revised the manuscript critically, and read and approved the final version.

## Pre-publication history

The pre-publication history for this paper can be accessed here:

http://www.biomedcentral.com/1472-6874/14/95/prepub
